# Multi‐genic pattern found in rare type of hypopituitarism: a whole‐exome sequencing study of Han Chinese with pituitary stalk interruption syndrome

**DOI:** 10.1111/jcmm.13272

**Published:** 2017-07-14

**Authors:** Qing‐Hua Guo, Cheng‐Zhi Wang, Zhi‐Qiang Wu, Yan Qin, Bai‐Yu Han, An‐Ping Wang, Bao‐An Wang, Jing‐Tao Dou, Xiao‐Sheng Wu, Yi‐Ming Mu

**Affiliations:** ^1^ Department of Endocrinology Chinese PLA General Hospital Beijing China; ^2^ Department of Endocrinology Hainan Branch of Chinese PLA General Hospital Sanya Hainan China; ^3^ Department of Molecular Biology Institute of Basic Medicine Chinese PLA General Hospital Beijing China; ^4^ Department of Endocrinology The First Affiliated Hospital of Xinxiang Medical University Weihui City Henan China; ^5^ Department of Endocrinology and Metabolism The 264 Hospital of PLA Taiyuan Shanxi China; ^6^ Department of Biochemistry and Molecular Biology Mayo Clinic Rochester MN USA; ^7^ Department of Immunology Mayo Clinic Rochester MN USA

**Keywords:** pituitary stalk interruption syndrome, whole‐exome sequencing, pathogenesis, pathway, bioinformatics

## Abstract

Pituitary stalk interruption syndrome (PSIS) is a rare type of hypopituitarism manifesting various degrees of pituitary hormone deficiency. Although mutations have been identified in some familial cases, the underpinning mechanisms of sporadic patients with PSIS who are in a vast majority remain elusive, necessitating a comprehensive study using systemic approaches. We postulate that other genetic mechanisms may be responsible for the sporadic PSIS. To test this hypothesis, we conducted a study in 24 patients with PSIS of Han Chinese with no family history using whole‐exome sequencing (WES) and bioinformatic analysis. We identified a group of heterozygous mutations in 92% (22 of 24) of the patients, and these genes are mostly associated with Notch, Shh, Wnt signalling pathways. Importantly, 83% (20 of 24) of the patients had more than one mutation in those pathways suggesting synergy of compound mutations underpin the pathogenesis of sporadic PSIS.

## Introduction

Hypopituitarism refers to deficiency of one or more hormones produced by the anterior pituitary or released from the posterior pituitary, the most common cause in adulthood is a pituitary adenoma, or treatment with pituitary surgery or radiotherapy 1. Pituitary stalk interruption syndrome (PSIS) is a rare type of hypopituitarism due to the blocked transportation for hormones from hypothalamus to pituitary, its estimated incidence of 0.5/100,000 births 2, 3. Patients with PSIS manifest various symptoms of pituitary hormone deficiency including growth retardation and infertility. The typical feature on MRI is the triad of a very thin or absent pituitary stalk, an ectopic posterior pituitary and hypoplasia or aplasia of the anterior pituitary gland 4, 5. The diagnosis of PSIS is largely based on MRI imaging together with clinical and laboratory findings.

Despite of extensive studies in past decades, the aetiology of PSIS still remains unclear. Perinatal events were once thought to be involved due to their significant prevalence within this group of patients. Subsequent studies on patients born to consanguineous parents revealed that germline mutations in genes including *HESX1, LHX4, OTX2, SOX, TGIF, PROP1* and *PROKR2* may be responsible for the defect 6, 7, 8, 9, 10, 11, 12, 13. However, those mutations were only identified in a few isolated familial cases, and molecular defects in sporadic patients who account for vast majority remain undetermined. In addition to sporadic PSIS, a small subset of patients with Fanconi anaemia may also exhibit underdeveloped or no pituitary gland manifesting PSIS. It is not known whether mutations in Fanconi anaemia genes *FANCA, FANCC, FANCG* or *FANCD* would directly be involved in the development of pituitary gland 14, 15. Taking together, little is known about molecular pathogenesis of sporadic PSIS. This has largely been due to the rarity of this defect and due to the lack of proper tools for systemic study.

In consideration of ethnic differences, as well as the difference between familial (with germline mutations) *versus* sporadic patients, we previously screened 33 Chinese non‐familial patients with PSIS for mutations in genes *HESX1, LHX4, PROP1, OTX2* and *SOX3* genes which were known to associated with familial PSIS 9, 16, 17. Only was a novel heterozygous sequence variant found in *HESX1* along with a few polymorphisms found in *LHX4* and *SOX3*. No *OTX2* abnormality was detected in our patients, suggesting that other pathogenic mechanisms are at play.

As patients with PSIS often exhibit different degrees of anterior pituitary hormone deficiency, ranging from isolated GH deficiency to combined pituitary hormone deficiency, it is possible that such a wide spectrum of phenotypes may be caused by mutations in different genes. Comprehensive identification of such a wide spectrum of defects requires the use of systemic approaches.

To this end, we performed whole‐exome sequencing (WES) on 24 patients with no family history of PSIS for mutations in protein‐coding genes and found that the most of sporadic PSIS patients in our cohort possess one or more mutations in genes associated with Notch, Shh and Wnt pathways.

## Materials and methods

### Patients

We collected 24 cases of confirmed PSIS between May 2004 and March 2014 from 251 hospitalized hypopituitarism patients in the Chinese PLA General Hospital, and the complete demographics of the patients are summarized in Table 1. All diagnosis was made by MRI showing the absence of pituitary stalk, defective anterior pituitary function and typical clinical features. None of these patients had any evidence of family history nor was born to consanguineous parents. Whole‐exome sequencing was conducted to a group of randomly chosen 24 typical patients with PSIS. This study followed the tenets of the Declaration of Helsinki and was approved by the Ethics Committee of the Chinese PLA General Hospital. The methods were carried out in accordance with the approved guidelines. Written informed consents from all participating patients were obtained prior to their participation in the study.

**Table 1:** Demographics and clinical features of patients with PSIS included in this study

Baseline	(*n* = 24)
Age (year)	25.0 (11.0–35.0)
Sex (male/female)	22/2
Familial history	0
Height (SDS)	−2.8 (−5.0 to −0.2)
Bone age (SDS)	−1.3 (−5.0 to 0.5)
Upper/lower segment ratio	0.9 (0.9–1.0)
Height to arm span ratio	1.0 (0.9–1.0)
Breech presentation (%)	45.8
Perinatal events (%)	37.5
GHD (%)	100
GHD+ACTH deficiency (%)	70.8
GHD+TSH deficiency (%)	54.1
GHD+LH/FSH deficiency (%)	70.8
GHD+ Hyperprolactinaemia (%)	12.5

GH deficiency(GHD) was confirmed when the peak GH values were less than 5 ng/ml (complete GH deficiency) and 10 ng/ml (partial GH deficiency), respectively, in pyridostigmine bromide test and insulin‐induced hypoglycaemia tolerance test (ITT). TSH deficiency was diagnosed if basal serum free T4 (FT4) was subnormal (<10.42 pmol/l) with an inappropriately low serum TSH concentration (<5.50 mU/l). ACTH deficiency was diagnosed by morning basal serum cortisol <198.7 nmol/l with no significant increase during hypoglycaemia. LH/FSH deficiency was diagnosed based on delayed or absent pubertal development with low serum levels of testosterone for males (<8.4 nmol/l) or oestradiol for females (<48.2 pmol/l) and blunted LH/FSH response to a GnRH stimulation test. Hyperprolactinaemia were defined as basal serum PRL higher than 17.7 μg/l for males and 29.2 μg/l for females, respectively.

SDS: standard deviation scores; GHD: growth hormone deficiency; ACTH: adrenal corticotropic hormone; TSH: thyroid‐stimulating hormone; LH: luteinizing hormone; FSH: follicle‐stimulating hormone.

### Whole‐exome sequencing and analysis

Genomic DNA was extracted from peripheral blood mononuclear cells from the patients in our discovery cohort. Exome capturing was performed to collect the whole exons of the human genomic DNA. The exon‐enriched DNA libraries were sequenced by 100 bp paired‐end reads on a HiSeq2000 sequencer (Illumina, San Diego, CA, USA). Typically, each sample gets a coverage of 100×.

Primary data came in fastq form after image analysis, and base calling was conducted using the Illumina Pipeline. All single‐nucleotide polymorphisms (SNPs) were identified using the NCBI dbSNP137, HapMap, 1000 human genome data set (20110521 release, http://www.1000genomes.org/) and an in‐house database of 200 Chinese healthy adults.

The genes harbouring non‐silent mutations or small InDels were clustered into functional groups using David bioinformatics tool (https://david.abcc.ncifcrf.gov). Enrichment analysis was carried out to reveal significantly enriched pathways.

### Data collection from online database and gene expression analysis

To verify whether the genes with our newly identified mutations are indeed expressed in pituitary gland/CNS/hypothalamus, we searched the Gene Expression Omnibus (GEO database ID: 105193902, 105209619, 105218156, 105228744, http://www.ncbi.nim.nih.gov/geo). We summarized their expression profiles in Figure 4.

## Results

### General features

**Figure 1:**
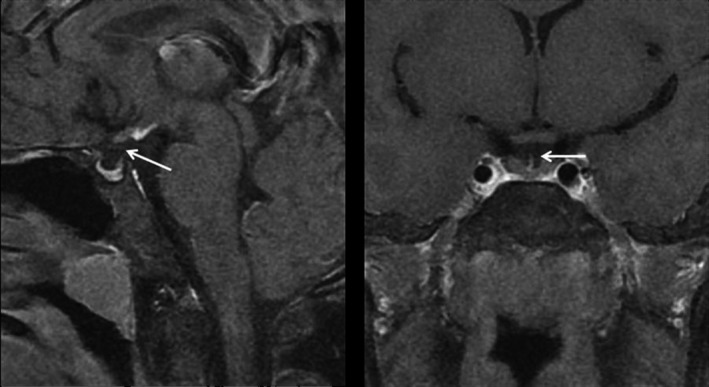
The sagittal and coronal pituitary on MRI. Left panel: The sagittal image showing the ectopic pituitary located at the floor of the third ventricle, along with a small anterior pituitary gland. Right panel: The coronal image showing the absence of pituitary stalk.

The general features of patients included in this study are summarized in Table 1. Specifically, the ratio of male to female was 11:1 among our 24 participants. Sex chromosome findings matched gender phenotype. Their average age was 25 years ranging from 11 to 35 years. There was no evidence of consanguineous parents and familial history in any of the cases. Eleven of these patients experienced breech delivery or footling delivery (11/24, 45.8%), while nine of them experienced some kinds of perinatal events including dystocia or suffocation (9/24, 37.5%). Only did two patients present midline abnormalities, one had partial absence of corpus callosum, the other suffered from Chiari‐I malformation and syringomyelia. A representative MRI image depicting abnormal pituitary development is shown in Figure 1.

### Molecular and *in silico* findings

Using WES, a total of about 5300 mutations were detected including about 4500 point mutations and 800 InDels (Data S1). The data were filtered with an in‐house SNP database of 200 healthy individuals of Han Chinese (provided by Beijing Genomics Institute) to exclude any ethnicity‐related common SNPs, and mutations were then sorted with PolyPhen2, a software that predicts possible functional relevance of mutations. We only selected for mutations with scores of 0.95 or higher meaning that they are likely missense mutations. This data sorting resulted in 275 likely relevant mutations in 270 genes (Data S2), in which none of previously reported gene mutations identified in familial patients with PSIS were found in our cohort.

**Figure 2:**
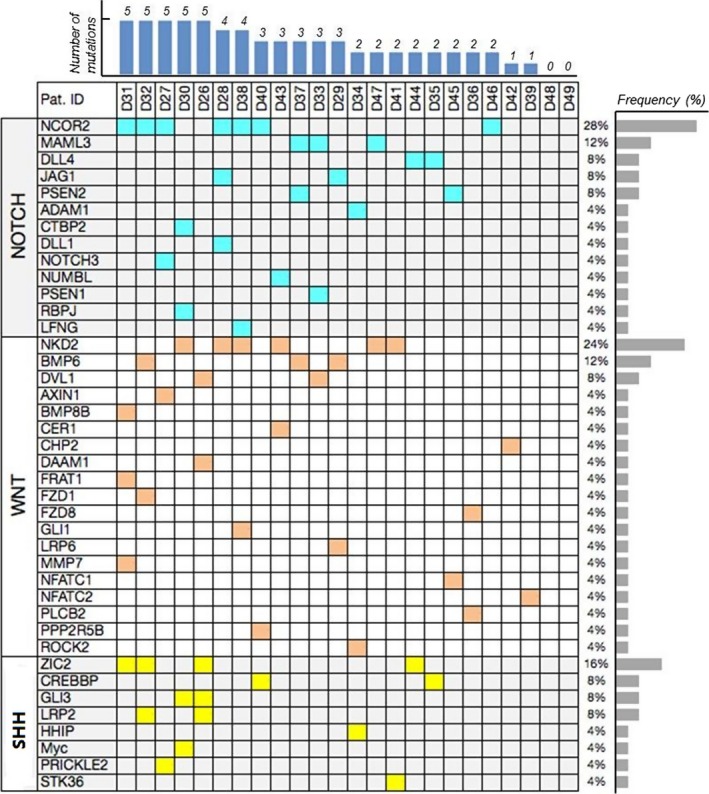
Gene categories in three pathways corresponding to each patient applied to WES. From left to right, mutations for each case were presented, and two of the participants did not show any mutations in the target pathways, thus only 22 cased were included. Red: WNT/β‐catenin signalling pathway; Blue: Notch signalling pathway; Yellow: Sonic hedgehog signalling pathway.

To better understand these mutations from their functional prospective, we further sorted the data using DAVID Bioinformatics Tool (https://david.ncifcrf.gov), a pathway enrichment analysis programme, and we identified 41 mutations in genes that are either the members of or associated with the Notch, Shh and Wnt signalling pathways (Fig. 2). Our results suggest that indeed mutations of a wide spectrum of and yet functionally related genes are involved in sporadic PSIS.

As all the mutations identified were heterozygous mutations which are likely to cause subtle changes in gene dose, and haploinsufficiency of these genes in mice is not known to cause any phenotypic defect in pituitary development, we have been suggested that each sporadic PSIS patient may possess mutations in multiple yet functionally related genes. For this reason, we tallied the number of mutations in genes associated with Notch, Shh and Wnt signalling pathways in each patient and found that 83% (20/24) of the patients had two or more these mutations, and some patients had up to five mutations in this group of genes (Data S3). Our data strongly suggest that compound mutations in multiple genes synergistically contribute the pituitary defects in sporadic PSIS.

Surprisingly, none of the previously identified mutations in genes *HESX1, LHX4, OTX2, SOX3* and *PROP1* from familial patients with PSIS were present in our data set, suggesting that familial and sporadic PSIS each may be driven by a unique set of gene mutations. Familial PSIS tends to have homozygous mutations in single genes leading to completely inactivation of their functions, while sporadic PSIS possess multiple heterozygous mutations in a set of related genes typically within Notch, Shh and Wnt pathways.

**Figure 3:**
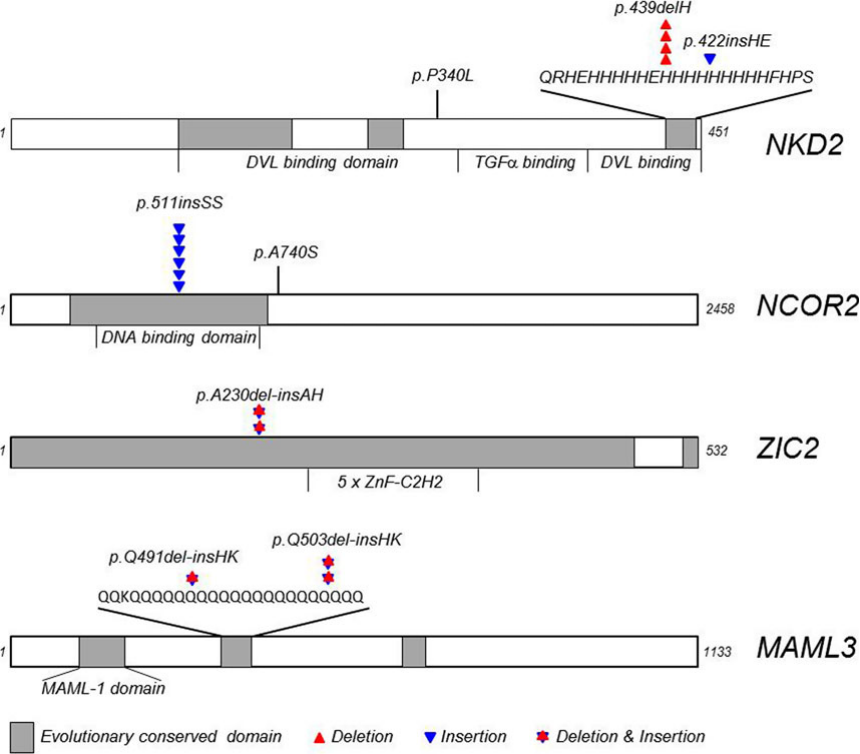
The location and their relations to conserved functional domains of mutations identified in this study. Different types of mutants were labelled as such.

Although mutations in multiple genes may impact the pathogenesis of PSIS, we suggest some of those genes may be more functionally important than others, and their mutations may be more frequently involved in sporadic PSIS. Therefore, we analysed mutation frequencies of these genes. Indeed, we found that *NCOR2* (of Notch pathway), *NKD2* (of Wnt pathway) and *ZIC2* (of Shh pathway) were mutated at the frequencies of 28%, 24% and 16%, respectively, significantly higher than that in other genes of the same pathway (Fig. 2), suggesting that these genes play more important roles in governing the well‐being of normal pituitary development. To further understand potential significance of mutations from their functional prospective, we aligned those mutations with the functional domain structure of those molecules, as shown in Figure 3, they are mostly localized to conserved functional domains, which further highlight their functional importance. By relating these mutations to published mutations in these genes in cancers (http://cancer.sanger.ac.uk/cosmic), we found that all these genes are tumour suppressor genes, implying that their mutations are likely to cause functional inactivation 18.

**Figure 4:**
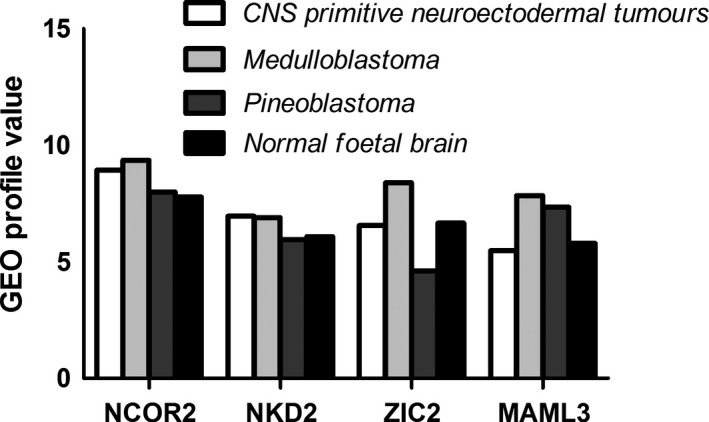
GEO profile value of NCOR2, NKD2, ZIC2 and MAML3 in humans.

Having identified mutations in a group of genes associated with Notch, Shh and Wnt pathways in sporadic PSIS patients, we then asked whether these genes are expressed in pituitary to functionally impact pituitary development. We surveyed previously published data in GEO database and found that *NCOR2, NKD2, ZIC2* and *MAML3* are indeed expressed in CNS/pituitary/hypothalamus (Fig. 4). Therefore, our newly identified mutations are likely to be the underpinning pathogenic mechanisms of sporadic PSIS.

## Discussion

Pituitary stalk interruption syndrome is a rare developmental pituitary defect, and germline homozygous mutations in certain transcription factors including *HESX1, LHX4, OTX2, SOX3, TGIF* and *PROP1* have been identified as the collective drivers for familial PSIS in several case reports 9, 13, 17, 19, 20, 21, 22, 23. Among these mutated genes, transcriptional factor *HESX1*‐mediated repression of Wnt/β‐catenin targets is required for the normal development of anterior forebrain 24; Wnt/β‐catenin signalling promotes midbrain dopaminergic progenitor specification, proliferation and neurogenesis by up‐regulating *OTX2* in progenitors 25; Notch signalling has been linked to *PROP1* expression 26; *GPR161* and *CDON*, the latest mutations found in patients with PSIS by WES recently, are regulators of Shh pathway 27, 28. Collectively, these pathways seem to be critical to pituitary development.

Reynaud *et al*. 6, 7 studied 83 patients with PSIS and found that only about 5% of the patients had identifiable mutations in this group of genes, and those 5% of the patients were exclusively familial cases including those born to consanguineous parents. Therefore, the vast majority (95%) of the patients are in fact sporadic of unknown causes. Efforts were also made previously to find genetic mutations in Chinese patients with PSIS in our laboratory, only were a few variants found in a small portion of patients 16. Although we had analysed all the reported genes that related with PSIS using Sanger sequencing in our Chinese patients and 100 healthy people, no significant results turned out after mutant frequency analysis (data not shown). Collectively, these findings suggest that sporadic PSIS may be caused by different genetic mechanisms.

To further answer this lingering question of what underpins the sporadic PSIS, in this study, we performed WES on blood DNA samples from 24 confirmed patients with PSIS of ethnic Chinese and uncovered that almost all sporadic PSIS patients possess multiple heterozygous mutations in genes associated with Notch, Shh and Wnt pathways. Furthermore, these heterozygous mutations are mostly localized to conserved functional domains, suggesting that they are likely to cause reduction in gene expression dose. Our findings demonstrated for the first time that the importance of these compound mutations in the development of sporadic PSIS likely through synergistic genetic interactions.

The paramount role of Notch, Shh and Wnt in embryogenesis is well documented. Their unique signals and concert actions are critical in controlling cell proliferation, differentiation, apoptosis, morphogenesis, embryo patterning and stemness. During pituitary development, Notch, Shh and Wnt signalling pathways are specifically and spatiotemporally expressed in pituitary tissues 21, 29, 30, 31, 32. Targeted disruption of some of the key components resulted in defective pituitary formation and/or function. However, those studies were conducted in mouse system. Here, we provide the first‐hand evidence showing this is also true in humans. Moreover, our data further highlight the importance of gene expression dose of these signalling pathways in the well‐being of pituitary development.

Among the most frequently mutated genes in our present study, *NKD2* is a negative regulator of Wnt/β‐catenin signalling and suppresses tumour growth and metastasis in osteosarcoma 33. Nuclear receptor corepressors (Ncors) are important for developmental and homoeostatic processes in vertebrates. *NCOR2* is always associated with tumour and haematopoiesis 34. Notch target gene expression is repressed by *RBPJ* through recruiting a corepressor complex, which includes *NCOR2*. Expression of Notch target genes is also reduced in mice lacking both *MAML1* and *MAML3*, two isoforms of Mastermind (Mam) which is essential for Notch signalling 35. The *ZIC* gene family encodes multi‐functional proteins essential for patterning and morphogenesis, mutations in individual *ZIC* genes resulted in diverse phenotypes such as cerebellar malformations. Recent studies suggest that the ZIC proteins can influence the transcriptional outcome of Sonic Hedgehog signalling 36. *ZIC2* is the only *ZIC* genes known to be associated with both major forms of holoprosencephaly (HPE): classic HPE and midline interhemispheric HPE 37, and it has been recently demonstrated to inhibit Wnt/β‐catenin protein signalling 38. Taking together, previous studies and our recent findings suggest that Notch, Shh and Wnt signalling pathways are indeed absolutely critical in the wellness of pituitary gland development.

As PSIS manifestation may also seen in a subset of patients with Fanconi anaemia, we carefully examined our clinical data, as well as our sequencing data, we found none of our patients showed any evidence of Fanconi anaemia, nor any mutations in Fanconi anaemia genes were present in our data set. It seems that PSIS in Fanconi anaemia is likely a separate entity with unique disease mechanisms.

In addition to the identification of mutations in these genes, we further provide evidence that *NCOR2, NKD2, ZIC2* and *MAML3* genes are likely to be expressed in pituitary gland as well as in brain and CNS tissues (Fig. 4). Therefore, our newly identified mutations are likely to be the underpinning mechanisms of sporadic PSIS.

Normality of our cells is maintained by the harmony of complexed genetic interactions, changes in expression of one or more genes leading to new biological equilibrium are the fundamental to all genetic diseases. It is therefore conceivable that single‐cause disease is rather rare while the concert action of multiple contingent factors may better explains many complexed conditions like PSIS 39.

## Conclusions

Compromised genetic interactions among multiple signalling pathways were likely to underpin the pathogenesis of sporadic PSIS. Further work is needed to clarify the inner relationship of the genes and pathways.

## Conflicts of interest

The authors confirm that there is no conflict of interests.

## Supporting information


**Data S1** Dominant results for 24 patients.

**Data S2** Mutations selected by polyphen2.

**Data S3** Muations in Notch, Shh, Wnt signaling pathways.
